# National Dental Association

**Published:** 1899-10

**Authors:** 


### Reports of Society Meetings.

### NATIONAL DENTAL ASSOCIATION.

(Continued from page 613.)

*Tuesday, August 1.—First Day.—Second Session.*

THE Association was called to order pursuant to adjournment, with the President occupying the chair.

On motion of Dr. Emma Eames Chase, the privilege of the floor was extended to Dr. Grevers, of Amsterdam, Holland.

The Virginia State Dental Society, through their representative, Dr. T. H. Paramore, tendered an invitation to the Association to hold their next annual session in Virginia, consideration of which was, on motion, laid over till the time for selection of place of meeting.

There being no further miscellaneous business, reports of sections were next in order.

Dr. S. H. Guilford, chairman of Section II.,—Dental Education, Literature, and Nomenclature,—read a brief report, which was referred to the Publication Committee.

The first paper presented by the section is entitled, "The Specialization of our Preliminary Education," by R. H. Hofheinz, Rochester, N. Y. A brief abstract is as follows:

After reviewing briefly what education was supposed to mean in the past,—the education of the Middle Ages,—designed to form either young monks or young knights, the conceptions of Erasmus, of Sturm, Comenius, and others, Dr. Hofheinz spoke of the illumination thrown upon the problem of education by the doctrine of evolution, and the importance of natural aptitudes, and of specialization. A one-sided scientific education may become as harmful to a profession, based upon æsthetic and artistic training, as a one-sided technical training would be. This is recognized in all dental schools,—but has it been recognized in the preliminary training of the dental student?

Are the three years of the dental college course sufficient to train unskilled hands that have had no previous chance for development?

Preliminary education is bestowed upon the mind, while the executive functions of the physical system are ignored.

A student who has not developed some manual skill, under favorable conditions, at the age of eighteen, does not possess the physical requisites for a good dental operation. The student who is not naturally endowed with a reasonable amount of technical ability will never become the dental operator so much needed. Hence the necessity of the introduction into all secondary schools of drawing and constructive work. This would be a new departure, but new only because the schools have not been doing their duty by the pupils intrusted to them. The educational value of manual training has been established beyond all contravention. It aims to develop in the pupil powers of thought and expression, and to call out the executive powers, and give self-confidence in dealing with actual material. To whom does this apply more than to the future student of dentistry? Many dentists have been complete failures, owing to a total lack of constructive ability. Many things have been written about that have never been done by the writers. Public education in art is needed; the art-spirit should enter into everyday labor. The public or primary school should take account of the æsthetic faculty which belongs to the higher nature of the child. The essayist quoted from a number of eminent educators in proof of the fact that manual training does not interfere with academic advancement; on the contrary, those who have followed the former courses show added intelligence in comprehending what is brought to their notice, and have a wider range of thought. Preliminary education must adjust itself to the needs of the future, and nowhere is this more necessary than with the future dentist. His early training must have a more specific direction,—not at the expense of science, but for its benefit. Hand in hand with the training of the mind must be that of the hand and eyes. The intellectual culture of constructive art is a great mental discipline, a great moral discipline, leading to more exact action, to greater honesty,—for honesty is but the moral expression of exactness.

DISCUSSION.

In the discussion of this paper

*Dr. Jas. Truman* said he was glad that some one had the moral courage to denounce the inartistic operations which confront us daily, and which, as the essayist has demonstrated, show a total lack of æsthetic culture. A gold crown on a front tooth best illustrates this point.

Another statement made by the essayist that deserves thought is, that a young man, who has attained the age of eighteen years without the opportunity of acquiring manual dexterity, will rarely or never acquire the practical skill essential to the successful dental operator. Dr. Truman said that he had held this view for many years. He would like those who believe that the dental student should take a full medical course, after his preliminary high-school and college training, before entering the dental college, to consider for a moment what would be the age of that young man on beginning the study of dentistry, and what would be the probabilities as to his attainment of manual dexterity at that age. Dr. Truman considered this the most impressive question suggested by the paper.

*Dr. E. V. Black* said that he wished to accentuate the thoughts expressed by Dr. Truman. He was most heartily in accord with the expression of the necessity for acquiring manual training in early school-life; of learning to use the hands; to accomplish something, to produce something, to be useful. With all our boasted advancement there is yet much to be gained in the future, especially in the direction of manual skill. To develop that will be of lasting benefit to the dental profession, the mind and hand working together for good, for that which shall endure.

As to "a gold crown on a front tooth," there is no more certain mark of mental debasement. The only wonder is that any man can find patients willing to submit to such disfigurement, or that any man who has sufficient aspiration to seek an education in dentistry, and who has the skill and taste necessary to accomplish such a work of art, can be so debased as to employ it in such a position.

*Dr. Geo. D. Sitherwood.*—It is a fact well known that there comes a time in the life of every individual when he cannot learn to write. It takes years to train the hand to form the letters mechanically, and the training must begin early. It is no less true of the training of the hand of the skilful dentist. There is a growing tendency to confine the study of dentistry to the dental college. Looking over the college announcements it will be seen that thirty or forty per cent. of the students have had no preceptor except "the faculty." They have had no preliminary training in a dental office, no opportunity of acquiring any manual skill previous to entering the dental college. It is time a halt was called in this direction. A graduate fresh from any college, no matter which, who has had only the opportunities offered during his college-life, will find himself deficient in skill when he undertakes his first piece of bridge-work, or his first crowns, if the faculty has been his only preceptor. They must have preliminary manual training before entering the dental college, and this should be acquired in the office of a competent preceptor.

*Dr. Truman W. Brophy.*—While I endorse much of what has been said by the preceding speakers, and heartily endorse the paper, I must dissent from the views of the last speaker, who apparently had in mind the conditions existing twenty years ago.

The young man who enters the dental college of to-day, where manual training is insisted upon, where such thorough, practical, technical work is done, can get as much benefit in three months in a dental school as he could do in a year in any dental office. The position taken by the last speaker is not well founded.

*Dr. C. N. Johnson* thinks there is danger in the increasing tendency towards specialization. Specialization tends towards narrow-mindedness. The mind should be broadened by culture, not narrowed by restricting it to a limited sphere. The man who works always with little things tends to become narrow-minded. The views of Dr. Sitherwood had his sympathy at one time, but the result depends largely on the preceptor, or on the college faculty. It is an actual fact that the first year of many a college student is spent in *unlearning* what he had acquired under a preceptor.

A visit to the dental schools, as they are now conducted, will correct many erroneous ideas on the subject of manual training. The older generation of dentists are usually sought as preceptors, but the methods in use in many such offices are not those taught in the dental colleges of to-day, but are rather those in vogue a quarter of a century ago.

*Dr. Sitherwood.*—I was discussing principles, not individuals.

*Dr. Garrett Newkirk.*—I was struck with the absolute correctness of the principle that unless a man develops mechanical skill early in life—before the age of eighteen, as was stated—he will not be successful in the manipulative work of operative dentistry. Let a farmer's boy, who has been holding the plough-handles till he is sixteen years old, attempt, at that age, to learn to write his own name, how slowly and laboriously he will form each letter. His fingers are stiff and the finger-tips are hard, and he grasps the pen-holder as he would a hoe-handle. Then see the student who has cultivated his abilities: the muscles so uncontrollable by the one are under absolute control of the other; the movements are rapid, almost mechanical. A man must have a disposition to handle tools or he will never succeed in the practical work of dentistry. It used to be the fashion to study under a preceptor, but now the colleges prefer to take a boy who has had no such preliminary work and start him at once in technical work. The advances made along this line have been a revelation to me. I would suggest that Dr. Sitherwood visit the schools and note the technical work.

*Dr. B. Holly Smith.*—There is no doubt but that the boy should start early in his life's work, if he would be skilled in manhood. There should be early opportunity for the development of mechanical skill; the mind and hand should be developed and trained together. The old preceptor-system was all right when the preceptor himself was all right. I have in mind one young man who had gone through the school's course, and whom, by courtesy, I took into my office, and who said that he learned more in three weeks by my chair than he had learned in the three years of college life,—he learned how to do things as he saw me doing them. But under the old system, when the student was relegated to the laboratory, when he had charge of the vulcanizer and collected bills, his chances were not very good of becoming a skilled operator.

*Dr. Hofheinz,* in closing the discussion, said that he had nothing to add to what he had said in his paper, but would repeat his conviction that manual training should go hand in hand with academic training; that one would not interfere with the other, but would be found a great help, broadening the mind, and quickening the understanding; giving a young man an early chance of choosing his life-work.

The second paper presented by Section II. was entitled "Dental History a Part of the College Curriculum," by B. J. Cigrand, Chicago, an abstract of which is given:

Evidence of the progress of modern dental science is found in the ever-increasing interest manifested in historical research.

Lessons of wisdom for the future are to be drawn from the events of the past.

To the dental student the history of the progress of dental art and science must be fraught with the deepest interest. Hence the value of the labors of those who have sought to gather the fragmentary knowledge of dentistry in the past and weave it into a true history of the science.

There are at present six medical colleges which include in their course of instruction the study of the subject of medical history. The Illinois school of dentistry was the first dental school to create a professorship in dental history. The Paris Exposition offers a most opportune time for presenting a full and complete story of the growth and development of the dental profession in America. The work, if it is to be done at all, must be done before the present generation fades away; before this century closes. Dr. Cigrand has delivered a course of lectures on dental history in several dental colleges, and has found that students were greatly interested in the subject.

DISCUSSION.

In the discussion of the subject

*Dr. Chas. McManus* expresed his gratification for the interest shown in the subject by the essayist. Although in some respects the subject is dry, it should be a matter of interest to all students and practitioners of dentistry, and he hopes the present agitation of the subject may yield satisfactory results.

*Dr. H. L. Ambler* believes that the subject of dental history, if properly put before the profession, is capable of doing great good. We clamor for a higher standard, but the only way to grow is to have a history of the past. The medical profession prides itself upon its past, and points to Hippocrates, Esculapius, etc. If we would put ourselves on the same plane, we must do all we can as individuals, as colleges, as State societies, as national societies, to have prepared a correct history of our past. This means a great deal of work. Let each and every one gather up all he can and put it in the hands of the committee appointed by this National Association. It can never be done as a private enterprise, as it will require an immense amount of money; it is a huge task. We want to call the attention of both the public and the profession to the fact that we have a history; that it runs back three thousand years, and that we are proud of it. It is a good idea to have it in the college curriculum, and I am glad to learn that a movement has been started in that direction. I think the time is ripe for all dental colleges to teach dental history, and to establish a chair upon this subject.

*Dr. W. C. Barrett.*—I am more than anxious to see a proper history of dentistry, but it must not include the many foolish traditions that have been handed down from the past, having no foundation in fact,—fables to please children. A proper history is a record of facts. The historian must be able to separate fact from fable. The truths of history must be taught in our schools.

*Dr. G. V. Black.*—In the medical schools medical history is taught in the fourth year. The students are referred to the collection of old books in which the record is to be found, and they are guided in their reading, but there is no one volume in which it is all written out. It is a study which offers many difficulties, but the reward is sure.

*Dr. Cigrand,* in closing the discussion, said that his paper was designed as an appeal to all to assist the committee by contributing such material as each might be able to gather. If each individual would feel that he had a personal interest in this matter, a great deal could be accomplished. We need a history, and we will have a history, though it may not be completed for years to come. The material is in existence, but much of it has yet to be discovered.

The third paper presented by the section is entitled "Dental Articulation and Occlusion," by Wm. Ernest Walker, Pass Christian, Miss.

The essayist urged the necessity for the continued use of the two words articulation and occlusion in denoting the relationship between the antagonizing surfaces of the teeth, restricting the use of the word occlusion to the indication of the positions of the teeth when the mouth is closed and at rest, the teeth coming together principally cusps to sulci. Articulation, as thus used, denotes the various relative positions assumed by the teeth in the lateral and protrusive excursions of the mandible. This interdental articulation is a very important consideration in the arrangement of cusps in prosthesis, in orthodontia, in contour operations, and in peridental diseases.

A change in the nomenclature of this subject has been suggested and recommended, restricting the use of the word articulation to its strictly anatomical sense,—the relationship of the teeth to their alveoli; the word occlusion to be made to do service in denoting not only a true occlusion ("Occlusion: a closing; a shutting up," *Century Dictionary*), but also all the phases of contact between the cusps of the upper and lower teeth in the various positions possible to the lower jaw.

That this use of the terms has not proved satisfactory to the profession at large was shown by the essayist, in the continued use of the word articulation with reference to the antagonizing surfaces of the teeth, by the best writers, not only in dental periodical literature, but especially by the authors of the different chapters in the "American Text-Book of Prosthetic Dentistry," quotations being given from the chapters by Drs. Essig, Burchard, Goddard, Molyneaux, Ambler Tees, W. W. Evans, and A. H. Thompson.

Its use was also shown by Dr. E. S. Talbot, in his latest work, "Interstitial Gingivitis." Its continued use by members of the Academy of Stomatology (Philadelphia), of the Institute of Stomatology (New York), of the American Academy of Dental Science (Boston), of the Section of Stomatology of the American Medical Association, of the New York Odontological Society, and numerous other scientific bodies, as shown by reference to page and volume of the leading dental periodicals published in 1898 and 1899.

The sense in which the essayist would use these two words is that given them by Dr. W. G. A. Bonwill, in his classic paper, "The Laws Governing the Articulation of the Human Teeth." As they have been so long used in this clearly defined and specific sense, it would seem advisable to continue to use them in that sense, at least until two equally distinctive but better terms shall be adopted, by which to indicate the radically different phases of relationship assumed by the teeth when at rest, and during the function of mastication.

It is always undesirable to make radical departures from long-established usage unless very decided advantages are apparent. The necessity for reference to the alveolar articulation of the teeth being of comparative rarity, the cumbrous, compound term "dento-alveolar articulation" might serve sufficiently well to indicate that relationship, the single word articulation applying to the contact of those surfaces with which we are most frequently concerned.

It is, however, greatly to be desired that proper discrimination be made by those who have occasionally used the terms articulation and occlusion as synonymous.

Discussion of this paper was deferred until after the reading of the fourth paper offered by the section, an interesting report, by Dr. J. A. Chapple, Atlanta, Ga., of a decision rendered by the Supreme Court of North Carolina in December, 1863: a decision by which the dental surgeon was declared the coequal, from a legal stand-point, with the physician. Owing to the lack of paper and other printing-materials at the time, and in the locality when and where this decision was rendered, it was never given newspaper publicity, and the record is to be found only in the annals of the Supreme Court of North Carolina.

This decision has a threefold significance:

1. That the dentist, in the full significance of the term, is a physician.

2. That, as such, according to Act of Congress, he is exempt from army service.

3. The physician under the law being exempt from jury duty, the dentist, being a physician, can also claim exemption, if it is not provided for by the respective States.

A fourth deduction is that the non-graduate dentist is not a physician, and cannot claim exemption under this decision, the judge having declared that "a regular graduated dentist is a physician." Dr. Chapple presented in full the decision "in the matter of Hunter," found in the 60th North Carolina Reports, Winston's, the decision having been given at Richmond Hill, December 3, 1863.

The question that arose was, Does the graduate dentist come under the definition of physician? The question being a new one, the case was adjourned and evidence taken as to the course of instruction in dental colleges, and the knowledge which it was necessary to acquire in order to obtain a diploma and practise with skill. The conculsion of the learned judge, from the depositions and the arguments filed, being found in the words: "I am satisfied that a regular graduated dentist is a 'physician.' . . . If a tooth has to be extracted, the 'surgeon dentist,' by his knowledge of 'physiology,' ascertains the condition of the system, and by his knowledge of 'materia medica' administers the necessary alteratives to put it in proper condition; by his knowledge of 'anatomy' he finds how the tooth is inserted in the jaw-bone, and knows what instrument will extract it with as little pain as possible and without injury to the bone, and the depositions state that frequently 'surgeon dentists' are called on to perform delicate operations on 'the facial parts' (the upper and lower jaw-bones), which require an intimate knowledge of the structure of the bones and the location of the arteries, veins, and nerves. In short, the teeth being more subject to decay and disease than any other part of the human body, I am satisfied not only that regular, educated dentists are 'physicians,' but that the human family are much indebted to them for confining themselves to a 'specialty,'—that is, one branch of the profession, whereby that which was some years ago a mere mechanical art, has become a useful and important science. It is, therefore, considered by me that John W. Hunter be forthwith discharged, with leave to go wherever he will."

There was no discussion of this paper, and after a partial report from the Committee on Credentials, showing the registration of thirty-eight delegates, the Association adjourned to nine A.M.

The presentation, by Dr. Truman W. Brophy, of patients from Chicago, who had been operated upon at a very early age for cleft palate and harelip, was made the special order of business for eleven A.M., Wednesday.

(To be continued.)

